# A high throughput assay of lichenase activity with Congo red dye in plants

**DOI:** 10.1186/s13007-021-00801-x

**Published:** 2021-10-09

**Authors:** Alexander A. Tyurin, Aleksandra V. Suhorukova, Igor V. Deineko, Olga S. Pavlenko, Viktoriia A. Fridman, Irina V. Goldenkova-Pavlova

**Affiliations:** Laboratory of Functional Genomics, Timiryazev Institute of Plant Physiology, Russian Academy of Sciences, Moscow, Russia

**Keywords:** Lichenase, Congo red, Expression, Plant reporter, Reporter proteins, Quantitative expression

## Abstract

**Background:**

Since the beginning of the use of reporter proteins for expression analysis, a variety of approaches have been developed and proposed; both qualitative and quantitative. The lack of simple methods for direct observation of gene expression in living organisms makes it necessary to continue to propose new methods. In this work, we consider a method for the quantitative analysis of the expression of thermostable lichenase from *Clostridium thermocellum* used as a sensitive reporter protein.

**Results:**

In this study, we report the design a high throughput fluorometric method for quantification of thermostable lichenase *C. thermocellum* using Congo red and further experimental verification of its relevance and efficiency in assessment of the functional role of regulatory sequences in the plant cell.

**Conclusions:**

The specific interaction between the dye Congo red and $\beta$-d-glucans formed the background for designing a high-throughput fluorometric assay for quantification of *C. thermocellum* thermostable lichenase as a reporter protein for plants. This assay (i) makes it possible to precisely measure the amount of reporter protein in a plant sample; (ii) has shown a high sensitivity for quantification of thermostable lichenase; (iii) is more time- and cost-efficient as compared with the Somogyi–Nelson assay; and (iv) is to the least degree dependent on the presence of the tested buffer components as compared with the Somogyi–Nelson assay.

**Supplementary Information:**

The online version contains supplementary material available at 10.1186/s13007-021-00801-x.

## Background

Currently, the attention of researchers is focused on the insight into the control mechanisms underlying the homeostasis in living organisms under both normal and different stress conditions. This control is implemented at several levels, namely, chromatin organization, transcription, pre-mRNA processing and splicing, translation, stability of protein product, and protein modifications. The recent advent of new experimental methods, such as RNA-seq and polysome analysis, has allowed for accumulation of a vast body of omics data, making it possible to construct the lists of genes the expression of which at the stages of transcription and translation is induced or regulated by certain factors, in particular, environmental impacts. The insight into the molecular mechanisms underlying the regulation of biological processes in general and gene transcription or mRNA translation in particular, requires the knowledge about the regulatory contexts potentially involved in the intricate mechanisms of gene expression regulation as well as experimental verification of the functional roles of such regulatory contexts in the plant cell [1–4]. A large toolkit of methods is currently available to determine the changes at the level of gene transcription and mRNA translation; their principles, advantages, and disadvantages are disused in several reviews [3, 5]. However, these methods are in most cases applicable to detection of the general alterations in transcription and translation and, as a rule, they require much time, considerable amount of reagents, and special equipment. The strategy of reporter systems is mainly used when studying the structure–function characteristics of the sequences of research interest. Reporter genes code for the proteins that possess either some unique specific features or unique enzyme activities and, thus, can be easily isolated from the total set of intra- and extracellular proteins. Correspondingly, the strategy of reporter genes considerably enhances the performance of such studies since it is much easier to determine the protein product of a reporter gene as compared with a target gene of interest. Taking into account these advantages of reporter systems over the other methods for studying gene expression regulation, they are widely used to experimentally verify the gene promoter regions and nucleotide contexts in differentially translated transcripts [3]. Expression cassettes that carry the sequence of a reporter gene the expression of which is controlled by a particular regulatory region selected by researcher are constructed for such studies [3]. Several reporter systems are available that have shown good performance in the studies of potential regulatory sequences or nucleotide contexts modulating the transcription or translation in plant systems, in particular, $\beta$-glucuronidase (GUS) [6, 7], different variants of fluorescent proteins (for example, GFP and RFP), and thermostable lichenase (LicBM) [8]. Commercial substrates and kits are available for these reporter systems as well as the corresponding methods for quantifying the expression of these reporter genes. Note that each reporter system has its own limitations with respect to the quantification of the protein product of a reporter gene and, in particular, for the use of high-throughput approaches. Earlier, the thermostable lichenase–$\beta$-1,3-1,4-glucanase (lichenase) (endo-$\beta$-1,3;1,4-glucan-d-glycosyl hydrolase) EC 3.2.1.73 (P29716) of *Clostridium thermocellum*–was proposed as an efficient reporter for plant systems [8], with a number of advantages over other reporters [9]. The following features make thermostable lichenase an attractive for this role: (i) ability to retain its functional activity upon N- and C-terminal fusions [9]; (ii) outstanding thermostability and high specific activity that can be assessed by simple and sensitive methods both qualitatively and quantitatively; (iii) thermostable lichenase can be used for hosting proteins that are stable only within narrow pH and temperature ranges, because lichenase itself is active within wide pH and temperature ranges; (iv) high lichenase thermostability, as well as retention of enzymatic activity, after precipitation of non-target proteins with ethanol, can be used for rapid and economical purification of the target fusion proteins. In particular, it has been demonstrated earlier that up to 50% of contaminating proteins can be eliminated by incubation of protein lysates at 65 °C, while the proteins fused with lichenase remain preserved [10]. Lichenases strictly catalyze the endohydrolysis of the $\beta$-1,4-glycoside bond adjacent to 3-O-substituted glucose residue in the $\beta$-glucans containing $\beta$-1,3 and $\beta$-1,4 bonds, for example, $\beta$-1,3-1,4-glucans of cereals and lichenan, which are as a rule used as substrates for lichenase quantification. The activity and amount of lichenase are usually assayed according to reducing sugars with lichenan as a substrate either using 3,5-dinitrosalicylic acid (DNSA) [11] or the Somogyi–Nelson assay [12]. However, these methods have considerable limitations since they are (1) highly specific towards reducing sugars and sensitive to the compounds containing aldehyde groups, typically present in plant tissues (which can cause considerable errors in the measurement accuracy) and (2) too time-consuming and thus poorly applicable to high-throughput approaches. This essentially limits their use in functional annotation of plant regulatory sequences, namely, functional analysis of a large list of nucleotide contexts obtained by omics technologies. All this demonstrates the need in other approaches making it possible to quantify thermostable lichenase as a reporter protein. According to experimental data, Congo red (CR) is a supramolecular dye the molecules of which in water solutions interact to give “ribbon-like micelles” fluorescing with the maximum in the orange–red range (approximately 600 nm) [13]. As has been shown, CR interacts with $\beta$-d-glucans from various sources [14]; moreover, the colorimetric methods with CR have been developed for assaying $\beta$-1,3-d-glucans [14]. According to the available experimental data, the fluorescence of CR solutions considerably increases upon binding to soluble carboxymethyl cellulose (CMC) [13]. However, the literature lacks any data on the use of CR for quantitative assay of $\beta$-1,3-1,4-glucanase (lichenase) by fluorometry. Thus, the goal of this work was to design a high-throughput fluorometric method for quantification of thermostable *C. thermocellum* lichenase using CR and further experimental verification of its relevance and efficiency in assessment of the functional role of regulatory sequences in the plant cell.

## Materials and methods

### Bacterial strains

We used *E. coli* strain XL1-Blue (Stratagene, United States): recA1 endA1 gyrA96 thi-1 hsdR17 supE44 relA1 lac [F’ proAB lacIqZ M15 Tn10(TetR)]; *E. coli* strain BL21(DE3) (Novagene, United States) F– dcm ompT hsdS(rB- mB-) gal (DE3); and Agrobacterium tumefaciens strain GV3101.

### Reagents and buffers

CR was purchased from Aldrich Chemical Company. Inc., Milwaukee, WI, United States (97% purity) and lichenan, from Megazyme, Ireland. The solutions used for spectral measurements were prepared by dissolving CR and lichenan in ddH2O. The other used reagents were of analytical grade. The buffer for storing the enzyme preparation contains 20 mM Tris–HCl pH 7.4, 0.1 mM EDTA, 1 mM DTT, 200 μg/ml BSA, 50% glycerol, and 100 mM KCl and the buffer for the assay with CR, 62.5 mM NaAc pH 6.0, 100 mM KCl, 0.1 mM EDTA, and 200 μg/ml BSA.

### Spectral characterization of the fluorescence of lichenan–CR complex

Steady-state fluorescence measurements were taken using a Synergy H1 multi-mode microplate reader (BioTek, United States). The CR fluorescence spectrum was recorded in the range of 450–700 nm with the excitation wavelength of 550 nm or in the range of 300–620 with the emission wavelength of 620 nm. Lichenan was used at a concentration of 125 μg/mL (This choice is stipulated by the value is a half of the saturation value; unless otherwise specified) and CR solution, at a final concentration of 0.005%. The excitation/emission spectrum was recorded in a Synergy H1 in 96-well microplates. The recorded spectra were compared to the blank reaction mixture containing deionized water instead of the sample.

### Expression and purification of thermostable lichenase

A overnight culture of the *E. coli* strain XL1-Blue carrying (transformed by chemical transformation) the earlier constructed plasmid pQE-LicB-M [15] was diluted (1:50) with LB medium (Amresco, United States) and grown to an OD600 of 0.5 at $37^{\circ }\hbox {C}$. Then, expression of the gene was induced with 1 mM isopropyl-$\beta$-D-1- thiogalactopyranoside (IPTG) to grow the culture at $28^{\circ }\hbox {C}$ for 48 h. The cells were separated from the medium by centrifugation (15 min, 3160*g*), washed twice with the buffer containing 50 mM Tris–HCl pH 8.0, and suspended in the buffer containing 50 mM Tris–HCl pH 8.0, 10 mM EDTA, 0.1% Triton X-100, 5 mM DTT, 0.01% SDS, and 10 mM NaCl. The cells were incubated at 65 °C for 30 min and clarified by centrifugation (30 min, 16 000*g*, 4 °C). The supernatant suspension was purified on a HisTrap HP column (GE Healthcare, 17-5247-01) according to the manufacturer’s protocol. The eluted proteins were dialyzed against 5 mM Tris–HCl pH 8.0 at 4 °C; the purified thermostable lichenase was dissolved in the buffer containing 20 mM Tris–HCl pH 7.4, 0.1 mM EDTA, 1 mM DTT, 200 μg/ml BSA, 50% glycerol, and 100 mM KCl to a final concentration of 1 μg/μL and used in the further experiments. The protein amount in preparations was measured with bicinchoninic acid assay (Sigma, United States) [16]. The protein preparations were separated by SDS-PAGE (12%) according to Laemmli [17]. Molecular weight of the proteins was assessed using Thermo Scientific PageRuler Unstained Protein Ladder (Thermo Fisher Scientific, Inc., United States).

### Quantitative determination of thermostable lichenase

The unit of Lichenase was determined using lichenan (125 μg/mL) as a substrate (incubation time, 20 min unless otherwise specified). The reducing sugars released from the substrate were determined using the Somogyi–Nelson assay [12]. Briefly, 40 μL of lichenase sample diluted with 0.05 M acetate buffer pH 5.0 to an analyzed concentration and 160 μL of the 0.125% lichenan solution prepared from 1% lichenan stock solution by diluting with 0.05 M acetate buffer pH 6.0 were quickly mixed to incubate at 65 °C for 20 min. Then, 200 μL of the Somogyi reagent was added to thoroughly mix the sample and incubate it in a boiling water bath for 40 min. The sample was then cooled to a room temperature, supplemented with 200 μL of the Nelson reagent, mixed, incubated for 10–15 min at a room temperature, and supplemented with 400 μL of acetone and 1000 μL of distilled water. The total volume of the reaction mixture was 2000 μL. The sample was thoroughly mixed and centrifuged for 1 min at 10 000 rpm in a (Centrifuge 5418R, Eppendorf) centrifuge. The OD of the sample was measured at 610 nm in a Synergy H1 using 96-well microplates (Corning). The measurements were performed against the control samples containing all components except for lichenase (enzyme background) and lichenan (substrate background), which were replaced with 40 μL of 0.05 M sodium acetate buffer pH 6.0. The content of reducing sugars was determined with a calibration plot constructed using glucose. The amount of enzyme that formed 1 μmol of reducing sugars (as an equivalent of glucose) over 1 min (equivalent to 1 mg of protein) was taken as activity unit. The design of the fluorometric assay for quantification of thermostable lichenase using CR is described in Results.

### Engineering of plant expression vectors

Standard molecular cloning procedures and PCR protocols were used. Restriction endonucleases, T4 DNA ligase, Taq and Pfu DNA polymerases, and phosphatases were used according to the manufacturer’s protocols (Promega, United States and Fermentas, Lithuania). The basic vector, designated as pLAUMe, was constructed in several stages. Initially, the *Sac*I/*Sma*I fragment carrying the reporter gene of thermostable lichenase, *lic*BM3, was cloned from the vector pQE-LicB-M [15] to the vector pPGG 1A [18] hydrolyzed with *Sac*I and *Sma*I; this gave the intermediate vector pPGG-L. The *Spe*I/*Xho*I fragment of pPGG-L carrying the reporter gene *lic*BM3 with an improved 35S RNA promoter and terminator of cauliflower mosaic virus (CaMV) was cloned to the vector pVIG-T [18] preliminary hydrolyzed with SacI and SmaI to get the intermediate vector pGLR. Then, the cassette pACT-uidA-Tnos was synthesized. This cassette comprised *A. thaliana* actin promoter, $\beta$-glucuronidase gene (*E. coli* *uid*A gene), and termination sequence of the *A. tumefaciens* nopaline synthase gene (the primers used for construction of this cassette are listed in Additional file 1: Table S1). The cassette pACT-uidA-Tnos was cloned into the vector pGLR preliminary hydrolyzed with *Xho*I to get the vector pLAUMe (Figure S1). The vector pLAUMe was used to construct the vectors pLAUMe-SynM, pLAUMe-GGR, pLAUMe-AT30, and pLAUMe-AT65 by inserting (via SLiC [19]) the regulatory sequences between CaMV 35S RNA promoter and the *lic*BM3 reporter gene sequence (Additional file 1: Table S1). The correctness of the fusion of the genes with the corresponding regulatory sequences in the plant expression vectors pLAUMe-SynM, pLAUMe-GGR, pLAUMe-AT30, and pLAUMe-AT65 was confirmed by sequencing.

### Plant material, agrobacterium strains, and agroinfiltration

*Nicotiana benthamiana* plants were hydroponically grown at a temperature of 22 ± 2 °C, a photoperiod of 8 h, and an illumination of 100 μmol quanta/(m2 s). Knop’s solution was used as a nutrient medium. The pLAUMe, pLAUMe-SynM, pLAUMe-GGR, pLAUMe-AT30, and pLAUMe-AT65 vectors were used to transform (by electroporation) the A. tumefaciens GV3101 strain as earlier described [20]. The transformed bacteria were selected on a medium containing kanamycin and designated according to the expression vector used for the transformation. The transformed agrobacteria were used for agroinfiltration into the abaxial epidermis of the leaves of 6-week-old *N. benthamiana* with a syringe without a needle as earlier described [21, 22]. After the agroinfiltration, the plants were cultivated under the same conditions for 7 days. Agroinfiltrated leaf fragments were used in subsequent analysis.

### Extraction of total soluble protein from plant tissue

For extraction of the total soluble protein, the plant tissue frozen in liquid nitrogen was ground in 0.05 M acetate buffer pH 6.0 (2–4 mL of the buffer per 1 g of plant tissue) and centrifuged at 10,000*g* for 10 min; the supernatant was collected for further experiments.

### Quantitative $\beta$-glucuronidase assay

$\beta$-Glucuronidase in plant extracts was quantified according to Jefferson et al. [6]. The amount of $\beta$-glucuronidase in the preparations was determined using a calibration plot and expressed in nanomoles (according to 4-MU) per unit volume per minute.

### Statistical analysis

All experiments were performed in at least eight–ten independent replicates. The data were processed using Statistica for Windows v. 9.0 and Microsoft Office Excel 2007 unless otherwise specified. The experimental measurements were made in five–ten analytical replicates. Arithmetic means and their standard errors are shown in the figures and tables unless otherwise specified.

## Results and discussion

### Lichenan–CR complex is assessable according to fluorescence intensity

**Fig. 1:**
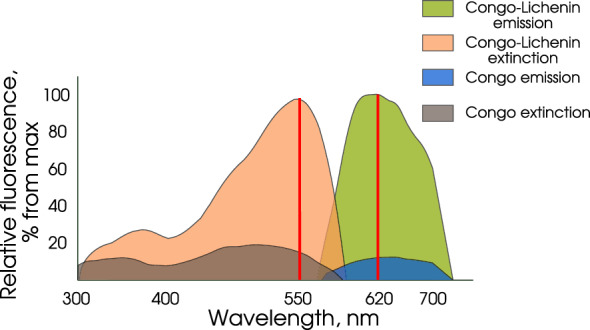
Fluorescence of Congo red (CR) solution and the CR–lichenan complex ($\lambda _{Ex}$ = 550 nm)

**Fig. 2:**
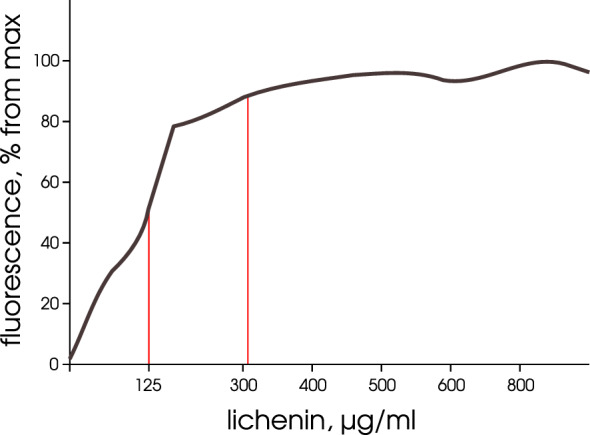
Fluorescence intensity of the CR–lichenan complex when using different lichenan concentrations: 0 to 300 μg/mL with an interval of 50 μg/mL and 300 to 900 μg/mL with an interval of 100 μg/mL

CR binds to polysaccharides, for example, to soluble carboxymethyl cellulose (CMC) or $\beta$-1,3-d-glucan [13] to give the complex the amount of which can be estimated according to fluorescence intensity. Moreover, CR does not require any specific binding sites in polysaccharides ($\beta$-glucans) [13]. According to earlier experimental data, CR binds to unhydrolyzed lichenan [23–25], which suggests that CR can form a complex with unhydrolyzed lichenan ($\beta$-1,3-1,4-glucan) and the amount of this complex is fluorometrically quantifiable. To confirm this assumption, we first recorded the fluorescence spectra of CR water solution (71.77 μM or 0.005%). The CR spectra were recorded in the range of 450–700 nm with an excitation wavelength of 550 nm or in the range of 300–620 nm at an emission wavelength of 620 nm. In addition, the spectra of CR water solutions supplemented with lichenan (at a concentration of 300 μg/mL) were recorded. The CR water solutions have a weak fluorescence intensity, which considerably increases with addition of lichenan, presumably, at the expense of CR–lichenan complex formation (Fig. 1). According to the fluorescence spectra, the emission maximum of the CR–lichenan complex is at 620 nm and the emission range, 580–750 nm. These data demonstrated the formation of this complex, which is detectable according to fluorescence intensity and allowed the parameters for assessing the fluorescence intensity of the complex to be determined: $\lambda$Ex = 550 nm and $\lambda _{Em}$ = 620 nm, which were used in the further analysis. Then we pinpointed the lichenan working range that would be optimal for the detection of CR–lichenan complex by fluorometry. The reaction mixture (final volume, 220 μL) comprised 0.1 V of CR solution (0.05%) and 0.9 V of lichenan solution at different concentrations obtained by dilution the stock solution of this substrate with the buffer for measuring lichenase activity (see "Materials and methods") in 96-well microplates. The changes in CR fluorescence spectra caused by its interaction with lichenan were recorded. A linear dependence of fluorescence intensity on lichenan concentration was observed for the lichenan concentration range of 50–200 μg/mL. The fluorescence intensity reaches a plateau with the increase in lichenan concentration to 300 μg/mL, which retained with the further increase in its concentration (Fig. 2). Presumably, this is explainable by the fact that when using a stationary CR concentration (0.005%) and a high lichenan concentration ($\le$300 μg/mL), only part of lichenan binds all CR molecules to form the CR–lichenan complex. These results allowed us to determine the optimal lichenan working range for the fluorometric assay, namely, 100–150 μg/mL, and a 125 μg/mL lichenan solution was used in the further analysis (Fig. 2).

**Fig. 3:**
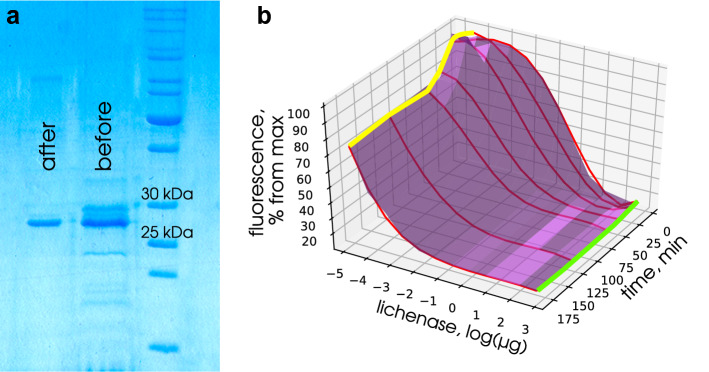
Fluorometric assessment of thermostable lichenase activity using Congo red. **a** Electrophoretic pattern of bacterial protein samples with thermostable lichenase before and after affinity chromatography purification; M, molecular weight marker (Thermo Scientific PageRuler Unstained Protein Ladder (Thermo Fisher Scientific, Inc., Waltham, Massachusetts, United States). **b** Comparative analysis of the fluorescence intensity of CR–lichenan complex depending on the lichenase amount in preparation and the time of incubation with the substrate (incubation time vector, 5, 10, 20, 40, 60, 120, and 180 min; protein (lichenase) amount vector, 16, 8, 4, 2, 1. 0.5, 0.25, 0.125, 0.0625, and 0.03125 μg/sample)

**Fig. 4:**
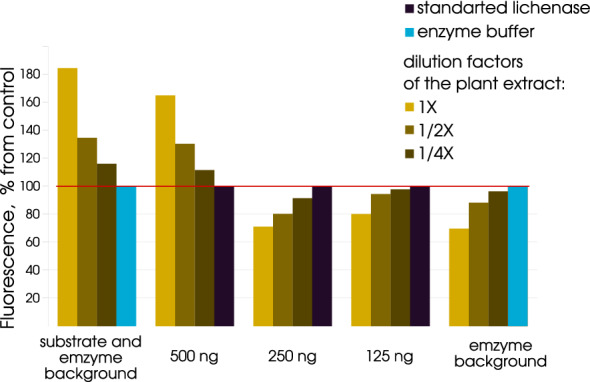
Comparative analysis of the fluorescence intensities of CR, CR–lichenan complex without lichenase hydrolysis, and after hydrolysis with different amounts of lichenase in the preparation depending on the presence of plant extracts

**Fig. 5:**
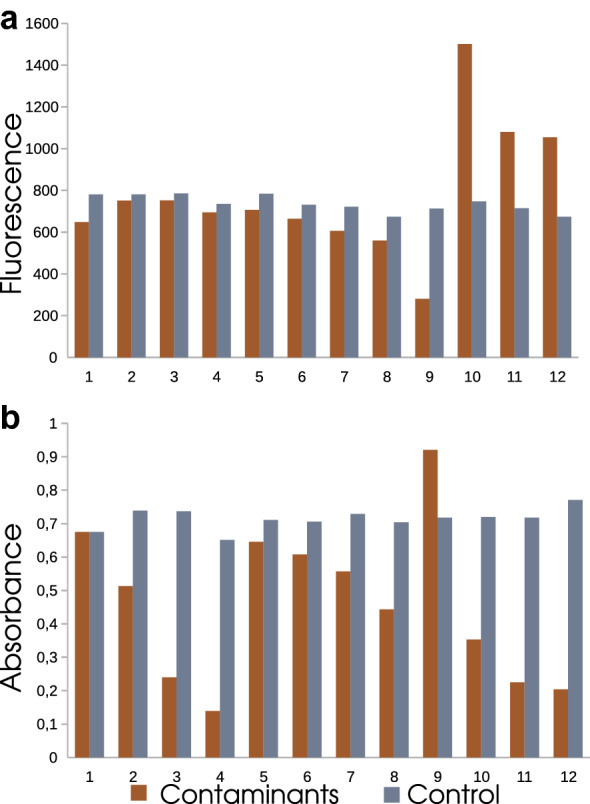
The effects of buffer components (EDTA, NaCl, and SDS) on the modulation of lichenase quantification in the samples by **a** fluorometric assay and **b** Somogyi–Nelson assay. EDTA, mM: 1 – 10, 2 – 20, 3 – 50, 4 – 100, NaCl, mM: 5 – 10, 6 – 20, 7 – 50, 8 – 100, SDS, %: 9 – 0.1, 10 – 0.2, 11 – 0.4, 12 – 0.5

**Fig. 6:**
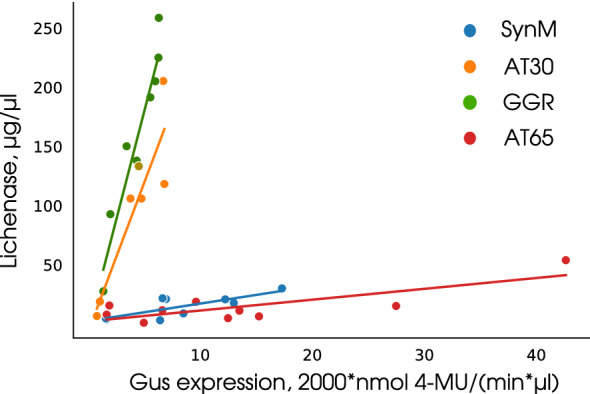
Comparative analysis of the fluorescence intensity of CR–lichenan complex in plant preparations after a transient expression of the vectors pLAUMe-GGR, pLAUMe-SynM, pLAUMe-AT30, and pLAUMe-AT65 in *Nicotiana benthamiana*. For glucuronidase, according to 4-MU and approximated with a logarithmic function of $\displaystyle f(x) = \ln (x + D) B + C$ type; for clarity, the glucuronidase activity was multiplied by 2000. The calibration curve for lichenase activity was constructed according to purified lichenase and smoothed with a hyperbolic function of $\displaystyle f(x)=D/(x*A+B)^C$ type. The results were normalized per volume taking into account the used dilutions

### Fluorometric quantification of thermostable lichenase using CR

The CR–lichenan complex was shown to display a high fluorescence intensity (Fig. 1) and this intensity strictly correlated with the amount on unhydrolyzed lichenan in the preparations (Fig. 2). Correspondingly, we assumed that the fluorometric assay of CR–lichenan complex was able to precisely determine thermostable lichenase as a reporter protein in the preparations using lichenan as a substrate. For this purpose, we produced a purified preparation of thermostable lichenase in *E. coli* with subsequent purification of the protein using affinity chromatography (Fig. 3a) and quantification of the target protein in the resulting preparation. The final preparation of thermostable lichenase was obtained by dissolving the protein in the enzyme storage buffer to a concentration of 0.4 μg/mL. The enzyme activity was verified using Somogyi–Nelson assay and amounted to 39 U per 1 mL of purified lichenase. Then, the dilutions of lichenase preparation were made so that the amount of lichenase in the tested samples varied from 16 to 0.03125 μg and a comparative analysis of the fluorescence intensity of the CR–lichenan complex versus the amount of lichenase in preparation and the time of its incubation with the substrate (5 to 180 min) was conducted. For this purpose, the reaction mixtures (final volume, 200 μL) containing 0.8 V of lichenan solution (125 μg/mL) and 0.8 V of lichenase preparation at tested concentrations were made by diluting the stock lichenan solution with the buffer for measuring lichenase activity (see Materials and methods). After the incubation of samples at 65 °C for the tested time intervals, the reaction of lichenan hydrolysis by thermostable lichenase was stopped by incubation in ice for 5 min to add 0.1 V of 0.05% CR (20 μL). The quantitative changes in the CR–lichenan fluorescence spectra in samples were recorded, i.e., the amount of lichenan that remained unhydrolyzed by the tested concentrations of thermostable lichenase depending on the time of lichenan–lichenase incubation was assessed. The reaction mixture was stained after the arrest of enzymatic reaction since CR partially inhibited the lichenase activity (data not shown). The following procedures were performed for the samples assayed by fluorometry: (i) correction for the CR noise, characterized by low fluorescence values, which could influence the measurement accuracy, and (ii) assessment of the maximum fluorescence of the CR–lichenan complex at the used lichenan concentration, which was necessary for further quantification of lichenase activity (level). Thus, blank samples were used for all measurements, namely, the samples containing (i) CR (dye background) and (ii) lichenan (125 μg/mL) + CR (substrate background). Thus, the measurements were performed against the control samples that contained all components except for lichenase (substrate background) and except for lichenan (dye background), which were replaced with the buffer for measuring the lichenase activity. The values of fluorescence intensity for the quantification of lichenase were calculated with the deduction of the dye and substrate backgrounds with the help of numerical analysis (see Materials and methods). The measurement results were compiled as a plot of dependence of fluorescence intensity on the natural logarithm of lichenase concentration (μg), shown in Fig. 3b. We recorded the changes (decrease) in the fluorescence intensity of the CR–lichenan complex after the incubation of lichenan with thermostable lichenase, which correlates with both the amount of lichenase in the preparation and the time of lichenase–lichenan incubation. A decrease in the fluorescence of CR–lichenan complex may be associated with a decrease in the amount of unhydrolyzed lichenan caused by thermostable lichenase, which presumably results from a decrease in the length of lichenan polymeric moiety during lichenase hydrolysis. In this process, a decrease in the CR–lichenan fluorescence correlated with an increase in the amount of lichenase in the tested preparation (Fig. 3b). In addition, the amount of lichenase in the preparation can be determined by varying the incubation time with the substrate. In particular, a 20-min incubation of lichenase with the substrate is optimal for the lichenase amount of 16 to 1 μg; 40-min incubation, for 0.5 to 0.125 μg; and 180-min incubation, for 0.0625 to 0.03125 μg (Fig. 3b). Thus, our data demonstrate feasibility of precise quantification of thermostable lichenase as a reporter protein using a fluorometric assay of the CR–lichenan complex.

A set of experiments was performed to clarify the potential applicability and efficiency of fluorometry for quantifying lichenase in plant preparations utilizing the modulation of fluorescence intensity of the CR–lichenan complex. In the first series of experiments, we assessed the possibility that plant extract components bound CR and, as a consequence, introduce noise to lichenase quantification in plant protein preparations via fluorescence intensity. For this purpose, we prepared plant extracts and their twofold and fourfold dilutions and estimated the fluorescence intensity in their interaction with CR at $\lambda _{Ex}$ = 550 nm and $\lambda _{Em}$ = 620 nm (Fig. 4; group of bars substrate and enzyme background). We observed that plant extracts increased the fluorescence intensity of CR water solution, which is most likely associated with the presence of the plant extract components that destabilized the CR supramolecular micelle when interacting with it. Presumably, these plant components are able to bind CR and, thus, cause dissociation of the CR supramolecular assembly, thereby increasing the fluorescence intensity [13, 14]. In this process, a decrease in the concentration of plant components by diluting (two- and fourfold) the plant extracts considerably decreases the CR fluorescence, making it almost comparable to the fluorescence intensity of CR water solution (Fig. 4; group of bars substrate and enzyme background). The second series of experiments was aimed at clarification of whether the plant extract components could influence the interaction of lichenan and CR and, therefore, modulate the fluorescence intensity of the formed CR–lichenan complex. For this purpose, we used plant extracts and their two- and fourfold dilutions, which were added to the samples containing CR (0.005%) and lichenan (125 μg/mL) water solutions. We analyzed the fluorescence intensity of the CR–lichenan complex ($\lambda _{Ex}$ = 550 nm and $\lambda _{Em}$ = 620 nm) in these samples (Fig. 4; group of bars enzyme background). This analysis demonstrated that the presence of plant extract (native) decreased the fluorescence intensity of CR–lichenan complex in a statistically significant manner. Note that the samples used in the second series of experiments contain lichenan unlike the corresponding samples in the first series, containing only CR. Note that the differences in the fluorescence intensity can be leveled by their dilution with the buffer and that a fourfold dilution of the plant extracts does not dramatically decrease this intensity. These data suggest that some components of plant extracts compete with unhydrolyzed lichenan for the interaction with CR, thereby interfering with the formation of both the complexes CR–lichenan and CR–plant extract components. We recommend choosing the appropriate conditions for each new experiment. The use of blank samples without enzymatic activity also minimizes the effect of background fluorescence on measurement accuracy. The third series of experiments was aimed at assessment of the potential modulation of the CR–lichenan complex formation in the process of hydrolysis by lichenase of its substrate (lichenan) depending on the amount of lichenase (500, 250, and 125 ng) in the analyzed preparation and the presence of plant extracts (Fig. 4; group of bars 500, 250, and 125 ng). As was observed, the modulation of fluorescence intensity depends on both the amount of lichenase used to hydrolyze lichenan in the analyzed preparation and the used plant extract (native or two- or fourfold diluted). In particular, 500 ng of lichenase used to hydrolyze lichenan in the presence of plant extracts in general led to an increase in the fluorescence intensity of the CR–lichenan complex as compared with the sample without the plant extract. However, the differences in the fluorescence intensity are leveled by dilution of plant extracts with the buffer and a fourfold dilution of the plant extracts fails to induce a statistically significant increase in the characteristic similar to the preparations containing only plant extracts and CR (Fig. 4; group of bars substrate and enzyme background). The potential mechanism of the observed modulation may be determined by a considerable decrease in the amount of unhydrolyzed lichenan caused by lichenase; on this background, the competition between the unhydrolyzed lichenan and plant extract components for the interaction with CR decreases and, as a consequence, both the remaining unhydrolyzed lichenan and plant extract components interact with CR, eventually leading to an increase in fluorescence intensity. In the case when a smaller amount of lichenase (250 and 125 ng) is used to hydrolyze lichenan in the presence of plant extracts, an opposite pattern is observed, namely, the fluorescence intensity decreases and this intensity also depends on both the used plant extract (native or diluted) and the amount of tested lichenase. The fluorescence intensity is to a lesser degree modulated when using a smaller amount of lichenase for lichenan hydrolysis as well as with a fourfold diluted plant extract. This may be determined by the competition of plant extract components and unhydrolyzed lichenan for the interaction with the dye (CR), which interferes with the formation of their complexes with CR so that only a small part of the dye forms the complexes, while the unbound molecules are responsible for a low fluorescence intensity. Thus, our data demonstrate the feasibility of an accurate fluorometry-based quantification of thermostable lichenase as a reporter protein in plant extract.

### Comparative analysis of thermostable lichenase quantification with Somogyi–Nelson and fluorometric assays

The following experiment was performed to find out whether the Somogyi–Nelson and fluorometric assays for the CR–lichenan complex were comparable in the sensitivity of lichenase quantification. We assessed the enzyme activity (amount) of lichenase in the tested preparations with a final enzyme amount of 16 to 0.03125 μ (20 min incubation of the enzyme at a temperature of 65 °C with the substrate; Additional file 1: Table S1). Note that the relevant data when quantifying lichenase amount using the Somogyi–Nelson assay can be obtained with the enzyme amount in the tested preparation above 0.5 μg under the specified incubation conditions since the optical density value after measuring the lichenase amount minus the substrate and enzyme backgrounds must fall into the range of 0.04–0.09. Thus, our data demonstrate a higher sensitivity of the fluorometric assay for lichenase quantification (15 ng in the preparation over 180-min incubation of lichenase with the substrate) as compared with the Somogyi–Nelson assay (to 2 μg in the preparation). As has been earlier demonstrated, thermostable lichenase is rather resistant to various chemical reagents, retaining a high level of enzyme activity in the presence of EDTA, SDS, Triton X-100, and several others [9]. In addition, we compared the effects of several components contained in the buffers for extraction of soluble plant proteins on the modulation of lichenase quantification in samples using fluorometric and Somogyi–Nelson assays, namely, EDTA (50–100 mM), NaCl (10–100 mM), and SDS (0.1–0.5%). For this purpose, the reaction mixtures (final volume, 200 μL) containing 0.8 V of lichenan solution (125 μg/mL) and 0.2 V of lichenase preparation (1 μg) were prepared by diluting the stock solution of the substrate in the buffer for measuring lichenase activity supplemented with one of the listed components in a tested concentration. After the incubation of the samples at 65 °C for 20 min, the hydrolysis was stopped by incubation in ice for 5 min. The half aliquots of each sample were (1) supplemented with 0.1 V of 0.05% CR solution or (2) successively supplemented with the reagents used for the Somogyi–Nelson assay followed by measuring fluorescence ($\lambda _{Ex}$ = 550 nm and $\lambda _{Em}$ = 620 nm) or optical density at 540 nm (Fig. 5). The modulation of fluorometric lichenase quantification in samples was observed only in the presence of SDS, i.e., under the conditions that can potentially weaken the CR–CR interaction. Presumably, the presence of SDS increases the dissociation of CR supramolecular assembly, which leads to an increase in the fluorescence intensity of the CR–lichenan complex in the corresponding samples. Note that the most pronounced increase in fluorescence is observable in the samples with 0.2% SDS, whereas 0.1% SDS causes an opposite effect, i.e., decreases the fluorescence of CR–lichenan complex, as well as the higher SDS concentrations of 0.4 and 0.5% (Fig. 5a). The mechanism of this pattern is vague and requires a separate study. All tested components of the buffers especially at elevated concentrations have a considerable effect on the lichenase quantification using the Somogyi–Nelson assay. Addition of EDTA (at concentrations of 20 to 100 mM) caused almost fivefold increase in lichenase activity; however, 10 mM EDTA had no effect on the measured value. The effect of SDS (0.2 to 0.5%) on the lichenase quantification using the Somogyi–Nelson assay is similar to that of EDTA, i.e., decreases the measured value of lichenase activity; however, 0.1% SDS displays an opposite effect, i.e., it increases the lichenase amount in a statistically significant manner. NaCl to a lesser degree influences the lichenase quantification; however, its effect was also observable at high concentrations (Fig. 5b). Thus, our data demonstrate that the lichenase fluorometric assay to the least degree depends on the presence of the tested buffer components as compared with the Somogyi–Nelson assay.

### Testing of fluorometric assay for quantification of lichenase in plants using transient expression technology

Transient gene expression in plants was used to test the fluorometric assay for lichenase quantification as an efficient technology for analyzing the functional role of regulatory sequences, which is time- and cost-efficient [26]. For this purpose, the basic vector pLAUMe was constructed. This vector carries two expression cassettes, one containing the reporter gene of thermostable lichenase with an improved cauliflower mosaic virus (CaMV) 35S RNA promoter and terminator and the other containing the reporter gene of $\beta$-glucuronidase with the A. thaliana actin promoter and the termination sequence of the nopaline synthase gene. The use of two expression cassettes in the vector is aimed at the leveling of noise sources when quantifying the level of reporter protein as the indicator of the functioning of the tested regulatory element, such as the species of the used plant, its age, and growth conditions, as well as the agrobacterial strain, including the density of the bacterial cell culture used for agroinfiltration. Note that pLAUMe also carries the gene for the tomato bushy stunt virus p19 protein, which acts as a suppressor of posttranscriptional gene silencing and has been specially designed for transient gene expression in plants [18]. The tested regulatory sequences (5’UTRs) were cloned into the 5–terminal region of the thermostable lichenase reporter gene vector pLAUMe (Additional file 2: Fig. S1); these sequences can potentially regulate the translation efficiency of reporter gene, thereby modulating the amount of reporter protein. The following regulatory sequences were selected for testing: GGR, the 5’UTR of A. thaliana geranyl-geranyl reductase gene (81 bp) [18]; SynM, modification synthetic enhancer (31 bp) [27]; AT30, a deletion variant of AT5G46430 5’UTR (22 bp) [28]; and AT65, a deletion variant of AT1G07260 5’UTR (22 bp) [28]. Molecular cloning procedures were used to construct the vectors pLAUMe-GGR, pLAUMe-SynM, pLAUMe-AT30, and pLAUMe-AT65; these vectors were transiently expressed in *N. benthamiana* model plants. The total soluble protein was extracted from agroinfiltrated plants to determine the amount of two reporter proteins, $\beta$-glucuronidase and thermostable lichenase. $\beta$-Glucuronidase was quantified as described in Materials and methods and lichenase, as described below. The reaction mixture (final volume, 200 μL) comprising 0.8 V of lichenan (125 μg/mL) and 0.2 V of soluble protein preparations extracted from plants was incubated at 65 °C for 20 min, stopped using the incubation in ice for 5 min, and supplemented with 10$\times$ CR solution to a concentration of 0.005% (20 μL of 0.05% solution). The samples were assayed for the fluorescence intensity of the CR–lichenan complex ($\lambda$Ex = 550 nm and $\lambda _{Em}$ = 620 nm). The fluorescence intensity values for quantification of lichenase were determined by deducting the dye and substrate backgrounds using numerical analysis. After eliminating the outliers, linear regression was calculated based on the amounts of $\beta$-glucuronidase (independent variable) and thermostable lichenase (dependent variable). The calibration curve for lichenase activity was constructed according to the purified lichenase and smoothed using the hyperbolic function of $\displaystyle f(x)=D/(x*A+B)^C$ type. The measured data were compiled as a plot; p and r values were calculated (Fig. 6). As is evident from the data, the measured lichenase amounts after eight–ten independent experiments for each vector strictly fit the corresponding regions (Fig. 6). The results confirm the hypothesis on a linear dependence between the lichenase amounts assessed in each sample. In general, these data suggest that the fluorometric assay based on the formation of CR–lichenan complex allows for an accurate quantification of the reporter protein in a plant sample, which is in particular, confirmed by the statistical criteria (p and r values). In addition, our results demonstrate that the tested GGR and AT30 regulatory sequences provide a more efficient mRNA translation of the thermostable lichenase reporter as compared with SynM and AT65 (Fig. 6).

## Conclusion

The specific features of the main properties of *C. thermocellum* thermostable lichenase, in particular, its outstanding stability, high specific activity, and thermostability, make this enzyme an attractive reporter system for studying various aspects in the mechanisms underlying gene regulation in plants as well as physiological roles of the target genes in heterologous gene expression in plants. The ability to refold in vitro after heat treatment at 70 °C or ethanol denaturation can be used for rapid and cost-efficient purification of plant protein lysates, in particular, when utilizing lichenase in plant biotechnology. For example, it has been shown that the heat treatment at 70 °C removes up to 50% of contaminating plant proteins, while lichenase and the proteins fused to lichenase remain intact [9, 10]. The *C. thermocellum* thermostable lichenase is active in a wide range of temperatures and pH [29], while the thermostable lichenase preparations isolated from biological samples, in particular, from plants, retain their high specific activity during a long-term storage in a container of a closed system under changing temperature or during a thawing–freezing procedure. The specific interaction between the dye Congo red and $\beta$-D-glucans formed the background for designing a high-throughput fluorometric assay for quantification of C. thermocellum thermostable lichenase as a reporter protein for plants. This assay (i) makes it possible to precisely measure the amount of reporter protein in a plant sample; (ii) has shown a high sensitivity for quantification of thermostable lichenase, allowing for detection of 15 ng of the protein over 180-min incubation of lichenase with its substrate versus the Somogyi–Nelson assay, able to detect to 2 μg in a sample; (iii) is more time- and cost-efficient as compared with the Somogyi–Nelson assay; and (iv) is to the least degree dependent on the presence of the tested buffer components as compared with the Somogyi–Nelson assay. In general, the results demonstrate that the fluorometric assay utilizing the formation of CR–lichenan complex allows for a precise quantification of the reporter protein in plant samples, which expands the range of application of C. thermocellum thermostable lichenase as a reporter protein for plants, in particular, for functional annotation of plant regulatory sequences, namely, functional analysis of a large list of nucleotide contexts obtained by omics technologies [3], as well as the use of this method in analysis of stable plant transformants [24, 30].

## Supplementary Information

**Additional file 1.** A table with description of plant expression vectors cloning.

**Additional file 2.** Map of the pLAUMe vector. *OCS* octopin synthase terminator, *p19* silencing supressor from tombusviruses, *TCTP* arabidopsis translationally controlled tumor protein promoter, *en35SCaMV* enchanced 35S CaMV promoter, *LicB* lichenase gene, *pAct* arabidopsis actin promoter, *uidA* gene of beta-glucuronidase, *T-Nos* nopaline synthase terminator, *CBCI* castor bean catalase intron.

## Data Availability

All datasets generated for this study are included in the paper/supplementary information.
