# Supplementary figures and images for: A high throughput assay of lichenase activity with Congo red dye in plants
Source: Plant Methods. 2021 Oct 9;17:102. doi: 10.1186/s13007-021-00801-x (PMC8501550; doi:10.1186/s13007-021-00801-x)

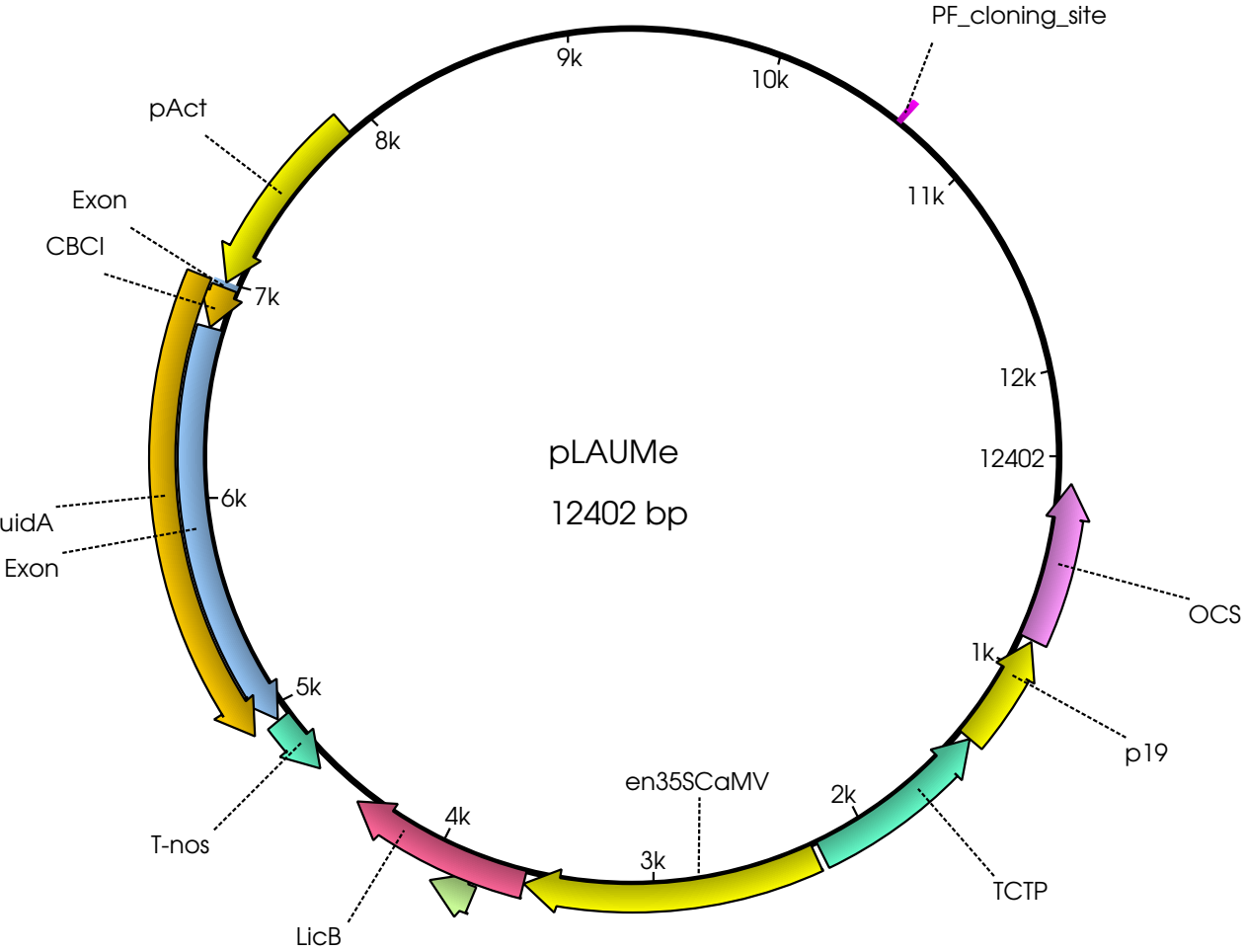

Supplement: Supplementary file 2 — Additional file 2. Map of the pLAUMe vector. OCS octopin synthase terminator, p19 silencing supressor from tombusviruses, TCTP arabidopsis translationally controlled tumor protein promoter, en35SCaMV enchanced 35S CaMV promoter, LicB lichenase gene, pAct arabidopsis actin promoter, uidA gene of beta-glucuronidase, T-Nos nopaline synthase terminator, CBCI castor bean catalase intron. [file 13007_2021_801_MOESM2_ESM.pdf]
